# Emerging biomarkers of prostate cancer (Review)

**DOI:** 10.3892/or.2012.1832

**Published:** 2012-05-25

**Authors:** SARAH K. MARTIN, TAYLOR B. VAUGHAN, TIMOTHY ATKINSON, HAINING ZHU, NATASHA KYPRIANOU

**Affiliations:** 1 Department of Molecular and Cellular Biochemistry, University of Kentucky College of Medicine, Lexington, KY, USA; 2 Division of Urology, Department of Surgery and Markey Cancer Center, University of Kentucky College of Medicine, Lexington, KY, USA

**Keywords:** prostate cancer, prostate specific antigen, biomarkers

## Abstract

Prostate cancer progression involves activation of signaling pathways controlling cell proliferation, apoptosis, anoikis, angiogenesis and metastasis. The current PSA-based test for the diagnosis of prostate cancer lacks sensitivity and specificity, resulting in missed diagnoses and unnecessary biopsies. Intense research efforts to identify serum and tissue biomarkers will expand the opportunities to understand the functional activation of cancer-related pathways and consequently lead to molecular therapeutic targeting towards inhibition of tumor growth. Current literature describes multiple biomarkers that indicate the properties of prostate cancer including its presence, stage, metastatic potential and prognosis. Used singly, assays detecting these biomarkers have their respective shortcomings. Several recent studies evaluating the clinical utilization of multiple markers show promising results in improving prostate cancer profiling. This review discusses the current understanding of biomarker signature cluster-based approaches for the diagnosis and therapeutic response of prostate cancer derived from panels of biomarker tests that provide a selective molecular signature characteristic of the tumor. As these signatures are robustly defined and their pathways are exhaustively dissected, prostate cancer can be more accurately diagnosed, characterized, staged and targeted with inhibitory antitumor agents. The growing promise surrounding the recent evidence in identifying and utilizing such biomarker panels, will lead to improvement in cancer prognosis and management of the therapeutic response of prostate cancer patients.

## 1. Introduction: the prostate cancer prediction challenge

Prostate cancer is the second leading cause of cancer-related death in men (1). With the current enhanced understanding of the molecular mechanisms leading to advanced metastatic disease, several factors present challenging obstacles in developing successful therapeutic modalities and screening tools for cancer detection and treatment (2). Malignant prostate cells progress through a series of genetic and epigenetic changes leading to aberrant proliferation, angiogenesis, evasion of apoptosis, metastasis to secondary sites and androgen independence (3). These pro-oncogenic pathways and key signaling molecules are currently being examined at the molecular and cellular level; with the application of this powerful technology in individual tumors, one would expect identification of novel markers indicating specific tumor properties in individual patients. A characterization of such biomarkers on a personalized level of analysis is expected to greatly impact the way physicians detect early prostate cancer and intervene to impair its progression to advanced disease.

Prostate cancer is characterized by distinct pathological changes indicating uncontrolled growth and biochemical emergence to androgen-independence. Consistent elevations in total prostate specific antigen (tPSA) in the serum, as well as marked decrease in apoptosis and tissue differentiation, are key factors in the progression of prostate tumors to advanced disease. Rigorous research efforts focused on androgen-independence and the determination of alternate androgen receptor (AR) pathways that cells employ to become androgen-independent are gradually adding to the current state of knowledge. With over 100 identified AR regulators, dozens of proposed androgen-independent receptor mutations, and several mechanisms of independence recognized including AR overexpression, local androgen production by the prostate and, proteolytic AR alteration to an androgen-independent isoform, a daunting task is evident (4).

In clinical practice, tPSA analysis has been the ‘gold’ standard in determining the presence and stage of prostate cancer. Accordingly, many clinicians recommend yearly serum tPSA and digital rectal examination (DRE) for men age 50 and older. As of 2009, the United States Preventative Services Task Force maintains that current evidence is insufficient to assess the balance of benefits and harms of screening for prostate cancer in men younger than 75 (I statement) and recommends against screening men age 75 and older (D statement) (5). PSA is a protein produced only by prostate tissue and would seem to be an ideal marker for prostatic disease. However, serum tPSA variability and its limited specificity to cancer are two properties currently limiting utility in prostate cancer screening and characterization (6). As varying serum tPSA values are found in patients with normal prostate function, benign prostate hyperplasia (BPH) and prostate cancer, high serum tPSA levels are not exclusive to the presence of prostate cancer (6) and this realization has drawn considerable controversy. Furthermore, while upward-trending tPSA values are often evident in individuals with progressive cancer, an absolute, linear relationship does not exist between serum tPSA and prostate cancer stage and metastasis (6). Despite finely orchestrated efforts in the clinical and translational setting, physicians and researchers have been unable to determine a standard tPSA level corresponding with precise disease staging, relying instead on tPSA cut-off values that vary among experts (7). In short, the path of disease progression results in a unique tPSA curve. This fact makes the tPSA test an indicator of predisposition to prostate abnormality rather than a definitive testing standard.

The challenge remains to define a firm biomarker level that definitively signals cancer initiation and/or progression to metastasis. Subsequently, other novel biomarkers are being studied for usefulness in diagnosing, staging and treating prostate cancer. Cancer is a disease of accumulating mutations causing uncontrolled cell growth with the contribution of epigenetic changes that can change the tumor phenotype. Uniformity is non-existant among each histologic cancer type and within each individual tumor. Thus, researchers have found other biomarkers associated with prostate cancer to be similarly variable as the disease progresses, limiting use in characterizing the disease (8). Examination of biomarker combination panels provides promise for early and precise prostate cancer diagnosis, and potential for the development of personalized treatments targeting the tumorigenic pathway defining individual tumors.

## 2. Serum biomarkers

Serum biomarkers are molecules produced by normal and abnormal cells. These molecules travel in blood plasma and are identified by serum assays. The most established and widely recognized serum biomarker for prostate cancer is total prostate-specific antigen (tPSA). PSA is a serine protease, also known as kallikrein 3, produced in an androgen-dependent manner by prostate ductal epithelial cells. PSA is generated by the healthy prostate at low levels, but can increase in association with cancer proliferation and prostatic disease progression (6). Currently, PSA is used to diagnose and stage prostate cancer, but has fallen under criticism with challenges to its sensitivity and specificity. In addition to PSA, several other prostate serum biomarkers have been studied (Table I)and their potential utilization is considered below.

### PSA

PSA is found in both serum and tissue however, total serum concentration, or tPSA, is most often used. While there is positive correlation between PSA and cancer progression, the correlation is not always consistent. Several variations of PSA have been studied including free-to-total PSA ratio, PSA density, PSA velocity and PSA isoforms. Free-to-total PSA ratio (or %free PSA) has been shown to increase the specificity for cancer diagnosis in patients with intermediate (4–10 ng/ml) tPSA values (9). This result is due to total PSA production increasing at a greater rate than that of free PSA in cancer patients, resulting in lower %free PSA. PSA density measures the tPSA per prostate volume. It relies on the premise that prostate cancer releases PSA into patient serum in greater proportion than the expected increase related to hyperplasia, resulting in greater PSA density in patients with prostate cancer vs. BPH. Based on reported results the validity of this measure has been debated (10,11). PSA velocity assesses the rate of PSA change over time. BPH yields a linear increase and cancer eventually results in an exponential increase (12). Problems with PSA velocity include poor sensitivity when initial PSA values are <4 ng/ml. Finally, different isoforms of both free and protein-bound PSA have been examined. Overall, sub-classifying the protein-bound isoforms has provided some advantage in distinguishing BPH from cancer; however, the effect is not greater than using %free PSA (13). In contrast, studies of free isoforms including BPH-specific PSA (BPSA) and pro-PSA (an inactive PSA precursor) have shown increased detection of clinically relevant cancers in patients with 2–10 ng/ml PSA and improved ability to differentiate from BPH (14).

### Kallikrein-4

KLK-4 is an androgen-dependent serine protease found in both serum and tumor tissue. Day *et al* demonstrated the elevated levels of anti-KLK-4 antibodies in sera of prostate cancer patients (15). Recently, KLK-4 has been implicated as a proliferative factor in prostate cancer cells and a potential mediator of the epithelial to mesenchymal transition. Ectopic expression of KLK-4 in prostate cancer cells increased the proliferation rate and motility of cells (16), while overexpression of KLK-4 resulted in a decrease of E-cadherin expression and increase of vimentin expression signaling, a potential EMT event (17). The trypsin-like activity of KLK-4 functions to activate pro-urokinase-type plasminogen into urokinase-type plasminogen activator (uPA), as discussed below (15). As more specific roles in prostate cancer development are elucidated for KLK-4, there is considerable promise that their ease of detection could effectively be utilized to diagnose and treat prostate cancer with a panel of other biomarkers.

### Steroid receptor coactivator-3

Src-3 (p/CIP, AIB1, ACR, RAC3, TRAM-1) is a 160-kDa protein and member of the Src family (16). Src-3 is a non-receptor tyrosine kinase which possesses an innate histone acetyltransferase activity as well as acting as a scaffold for recruitment of other coactivators to the transcription initiation complex (17). The recruitment of Src-3 to the PSA promoter in the presence of androgen and the physical interaction between the steroid receptor and Src-3 have been implicated in tumorigenesis (16,18). However, Src-3 overexpression is not unique to hormone-dependent cancers although it is well characterized in cancers of the breast, ovary, and prostate. Src-3 overexpression has been observed in gastric and pancreatic cancer, which suggests it may be facilitating tumorigenesis via other transcription factor interaction partners (16). The increased presence of Src-3 in serum samples has been correlated with enhanced cell proliferation and hormone-independence and inversely-related to cell apoptosis (17). In patients undergoing radical prostatectomy, PSA recurrence is an indicator of metastasis and disease progression; patients which scored higher on Src-3 overexpression were significantly more likely to undergo recurrence (16). Therefore, Src-3 serves as a viable indicator for disease recurrence. The ability of Src-3 inhibitors to impair prostate cancer progression and metastatic spread is currently being evaluated *in vivo*. Interestingly, a population based study of the polymorphic CAG/CAA repeat length in Src-3 gene has provided some preliminary evidence that a racial-associated prostate cancer risk may lie herein (19). Src-3 may hold the potential to serve as both a risk determinant and an indicator of recurrence.

### Minichromosome maintenance protein (Mcm5/7)

Minichromosome maintenance proteins are key players in the initiation of DNA replication and chromosomal duplication (20). Interestingly they are expressed in all phases of the cell division cycle, but silenced in phases in which growth is not occurring. With respect to prostate cancer, it has been shown that Mcm5 is overexpressed in prostate tissue and serves as an independent predictor of survival in patients undergoing radical prostatectomy, androgen deprivation therapy or radiotherapy (21). Contributing further to its potential as a biomarker, is evidence of the low levels of Mcm expression in normal and benign hyperplastic prostatic tissue. Recent work by Dudderidge *et al* revealed that Mcm5 levels are increased in urine sediments of patients with prostate cancer compared to those without and confirmed that Mcm5 levels are not increased in patients with BPH (21). While Mcm5’s role in prostate cancer detection and diagnosis is still currently being investigated, its usefulness on the development of a panel of biomarkers could be vital for the early detection of prostate cancer in the near future.

Mcm7 is another member of the proteins which together form a portion of the pre-replication complex which licenses DNA replication and is being investigated for its usefulness in identifying prostate cancer progression. An investigative comparison of Ki67 vs. Mcm7 immunohistochemistry staining was conducted and demonstrated that Mcm7 correlated highly with Ki67, but demonstrated an improved ability to distinguish between benign, PIN and adenocarcinoma (20). Further evaluation of Mcm7 expression with cancer progression, may prove the utility of this new marker.

### E-cadherin

E-cadherin is a major mediator of cell-cell adhesion junctions insuring communication between neighboring healthy cells and their connection to the surrounding extracellular matrix (ECM). Anoikis is a unique mode of programmed cell death consequential to loss of adhesion to neighboring cells and the ECM (22). The ability of prostate cancer cells to evade anoikis, and thus successfully invade and metastasize is driven by loss of E-cadherin expression and upregulation of epithelial-mesenchymal transition (EMT) regulators (22). Elevated levels of serum cleaved E-cadherin were demonstrated in metastatic prostate cancer cells, conferring the loss of the need for adherence to the surrounding ECM matrix and tissue (23). Furthermore, evidence has pointed to the switching of cadherin type expression with cancer progression. The loss of E-cadherin expression and gain of N-cadherin and cadherin-11 expression is seen in epithelial derived tumors (24). This cadherin switching has been associated with enhanced invasive capacity, metastasis, and dismal clinical outcomes; furthermore, it may serve as a pivotal biomarker of epithelial to mesenchymal transition.

Further evidence of this molecule’s therapeutic promise has been the recent use of small activating RNAs (saRNA) or non-coding, double stranded RNA molecules that can induce gene transcription by targeting promoter regions specific to the gene of interest. Through the use of saRNAs targeting E-cadherin expression, Mao *et al* demonstrated decreased cell migration and invasion of PC3 prostate cancer cells transfected with the E-cadherin specific saRNA (25). Thus, not only could the cleaved E-cadherin fragment be utilized as a promising marker of disease progression and metastasis, but it potentially could be targeted as an inhibitor of metastasis.

### Early prostate cancer antigen (EPCA-2)

Utilization of proteomics approaches has expedited the search for new biomarkers in cancer. Investigation into changes within the structural nuclear proteins have yielded identification of novel prostate cancer biomarkers (26). This characterized protein, EPCA-2, is elevated in sera of prostate cancer patients, but not in healthy patients. Subsequent studies have focused on raising antibodies against specific EPCA-2 epitopes that are both easily analyzed in serum, and specific to prostate cancer. The goal of these studies was to determine an effective screening tool for prostate cancer. One epitope, EPCA-2.19 shows considerably promise (27). An initial study from known samples determined a serum cut-off value of 0.5 ng/ml EPCA-2. A follow-up prospective study of 328 men showed that EPCA-2.19 has 94% specificity and 91% sensitivity in separating normal men and men with BPH from those with prostate cancer using the aforementioned cut-off of 0.5 ng/ml (27). Antibodies against another epitope of the same protein, EPCA-2.22, have furthermore been shown to distinguish organ-confined from non-organ-confined prostate cancer. One could envision how combination assays including both antigens might be applied for detection and staging of prostate cancer (27).

### Interleukin-6 (IL-6) and interleukin-6 receptors (IL-6R)

The cytokine interleukin-6 is most commonly known for its role in inflammation but has recently been evidenced for a role in the development of different cancers including prostate cancer. Elevated IL-6 and its soluble receptor have been linked to aggressive prostate cancer features including increased tumor volume, elevated overall Gleason score, distant metastases and decreased survival (28). *In vivo* studies have suggested a pathogenic role for the cytokine in prostate cancer and thus sparked new research involving the mechanisms of its effect (29). In order to exert its cellular effects, IL-6 must bind to the IL-6 receptor (IL-6R) to form a complex capable of binding to specific signal transducing proteins on the cell membrane. Two forms of the receptor exist, one being membrane bound (mIL-6R) and the other being soluble (sIL-6R). The soluble receptor isoform has been implicated as a predictor of metastatic disease. Its elevation along with the elevation of IL-6 has been demonstrated in patients who develop metastatic disease vs. patients who do not have disease recurrence within 5 years (29). It was further shown that sIL-6R compared to IL-6 demonstated a more robust correlation with disease progression (28). Recent work by Santer *et al* demonstrated increased cell motility and migration as well as decreased cell adhesion of prostate cancer cells in the presence of IL-6 with sIL-6R, but not IL-6 alone (30). Further understanding of the IL-6 pathway and the effect of its soluble receptor bound form will allow for more specific utilization of IL-6 as a marker of prostate cancer progression and metastasis. The incorporation of IL-6 and sIL-6R into a panel of preoperative blood based biomarkers improved the predictive capacity of the panel significantly in patients undergoing radical prostatectomy (28).

### Prostate cancer gene 3 (PCA3)

PCA3 is a prostate specific non-coding RNA which has been found to be highly overexpressed in >95% of primary prostate tumors, and furthermore, a 66-fold upregulation compared to adjacent non-cancer tissues (31). Through the intense contributions by Jack Schalken’s group, the recognition of this attractive new marker for prostate cancer shows considerable promise. Thus, the prostate cancer gene 3 (PCA3) assay has been argued in European and USA studies to better identify men at high risk of a positive biopsy and moreover to discriminate the best candidates for a repeat biopsy. Significantly enough, the probability of a positive repeat biopsy increased with increasing PCA3 score (31). In the clinical setting, the best diagnostic accuracy is potentially obtained in the ‘grey’ zone in which the yield of the free-to-total PSA ratio (f/tPSA) is maximal. Testing for elevated PCA3 has evolved into a quantitative urine test to facilitate prostate cancer diagnosis via non-invasive methodology (32). Comparison of the performance of PSA vs. PCA3 urine test was investigated by Roobol *et al* (33). They found that as a first line screening, PCA3 was an improved evaluative tool for performance characteristics and identification of serious disease in a prescreen population of 721 men (33). Based on the development of the clinical urine test and incorporation into many prostate cancer risk calculations, PCA3 is likely to become a lead biomarker.

## 3. Tissue biomarkers

Studies utilizing tissue specimens taken during diagnostic biopsy or radical prostatectomy, have shown that the expression of certain proteins, including uPA, enhancer of zeste homolog 2 (EZH2), prostate specific stem cell antigen (PSCA), androgen receptor (AR) and fatty acid synthase (FAS) correlates with tumor stage. The value of these molecules in the clinical arena is not limited to diagnosis, but many of these biomarkers produced could potentially be targeted to disrupt tumor progression to metastatic sites. The following tissue markers are currently being investigated for their clinical value in prostate cancer.

### Urokinase-type plasminogen activator

The activation of the uPA cascade via the interaction of the inactive precursor with a soluble or membrane bound uPA receptor (uPAR), results in extracellular matrix remodeling via degradation of the ECM and the basement membrane. The activation of a broad spectrum proteases by the uPA network facilitates metastasis of tumor cells and angiogenesis (28). Amplification of the *uPA* gene and increased *uPA* copy number have been described in patients with metastatic prostate cancer (34). This evidence supports a potential role for uPA as a molecular target for both early identification and inhibition of metastatic prostate cancer. Reported results established that uPA inhibition leads to a marked reduction in the invasive ability of prostate cancer cells (34). As described for IL-6/sIL-6R, the inclusion of uPA level in a preoperative blood based panel of biomarkers significantly enhanced the predictive power of the panel (28).

### Enhancer of zeste homolog 2

The pioneering work by Chinnaiyan’s group identified EZH2 protein in many human malignancies, including renal, breast and prostate cancer (35). The expression of this protein is associated with cancer metastases, localized to tumors with poor prognosis and found in combination with depressed E-cadherin expression and associated short term disease-free survival (36). EZH2 functions as a histone methyltransferase and its overexpression has been evidenced in castration-resistant, metastatic prostate cancer. Analysis by Li *et al* found that levels of EZH2 RNA and protein were significantly higher in prostate cancer cells than BPH or in human prostate intraepithelial neoplasia (HGPIN) (37). Also there was a significant increase in EZH2 in tumors with aGleason score >7 vs. <6 in addition to the significant positive correlation of EZH2 to TNM stage increasing with tumor progression (37). Further study into specific mechanisms of action of EZH2 have linked it with the gene fusion found in 50% of prostate cancers of TMPRSS2, an androgen-regulated gene, and the oncogenic ETS transcription factor ERG. ERG itself activates EZH2 allowing the methyltransferase to induce its repressive epigenetic agenda (38). The neuronal chemorepellant and tumor supressor gene SLIT2 has also been linked to EZH2. EZH2 targets SLIT2 and inhibits its expression (39). Levels of SLIT2 have been found to be downregulated in a majority of prostate cancers and a low level of SLIT2 has been associated with not only agressive prostate cancers, but breast and lung cancer as well (39). SLIT2 is downregulated via hypermethylation of the SLIT2 promoter accomplished by the enzymatically catalyzed actions of EZH2 on the lysine 27 of histone H3 (39).

*In vitro* studies have successfully shown the inhibition of prostate cancer cell proliferation using molecules targeting EZH2 (35). Recently, microRNA technology was effectively used to inhibit EZH2 expression resulting in a decreased migratory and invasive ability of prostate cancer cells (40). Although this pattern of overexpressed EZH2 and depressed SLIT2 is observed in other cancer types, the combination of these two may serve as a pertinent duo-panel of characteristic biomarkers for prostate cancer prognosis. Additional trials will enable the documentation of an association between elevated EZH2 with prostate tumor aggressiveness and low SLIT2 expression linked to poor prognosis (39).

### Prostate stem cell antigen (PSCA)

PSCA is a unique antigen found in prostate tissue, both healthy and diseased. In a recent study, PSCA was expressed in 94% of primary tumors and 100% of metastatic samples (41). Higher levels of PSCA were also significant in predicting an increase in cancer stage, Gleason score and androgen-independence. In another study, PSCA mRNA expression in tissue acquired from transurethral resection of the prostate (TURP) for BPH in patients with negative preoperative biopsy predicted subsequent cancer incidence (42). Therefore, PSCA serves as another potential therapeutic molecular target, as well as prognosticator of cancer incidence and progression.

### Androgen receptor (AR)

AR is a key protein functioning as a nuclear transcription factor in prostate cancer cells that may be used on a panel for prostate cancer screening. Prostate cancer progression is associated with acquisition of androgen-independence, resulting in metastatic lesions (4). Indeed, the emerging understanding of the mechanism of therapeutic failure of advanced prostate tumors involves upregulation of AR or activation of its transcriptional activity via a ligand-independent manner leading to castration-independent disease (43). Mechanisms of this development have been proposed including the development of an AR splice variant. Studies by Sun *et al* demonstrated a particular splice variant of AR found in humans whose transfection into mice led to castration-resistant prostate tumors and whose ratio to the full length androgen receptor positively correlated with castration resistant disease (44).

The relationship of the AR and its functionality to currently researched prostate cancer biomarkers is an area resounding with promise. A study by Dahlman *et al* studying the prostate cancer marker β-microseminoprotein (MSMB) has shown that its expression is in part controlled by androgen availability and a low level is associated with poor outcome and more aggressive disease (45). Further support for its inclusion in a biomarker panel is offered by the fact that MSMB expression was shown to be associated to high EZH2 expression and thus could be a possible target of epigenetic silencing effects (45). Consequently, the AR may serve not only as a therapeutic target, but also as a candidate for biomarker panels predicting prostate cancer metastasis, independent of androgens.

### Fatty acid synthase (FAS)

FAS is an androgen-regulated metabolic enzyme involved in *de novo* biosynthesis of fatty acids (46). *FAS* mRNA and FAS protein are both significantly overexpressed in prostate carcinomas (47). Furthermore, mRNA and protein levels were demonstrated to increase progressively with normal prostate to prostatic intraepithelial neoplasia, low grade, high grade and androgen-independent bone metastases (47). Based on these observations, FAS expression may be useful as a biomarker to assess disease staging and progression, especially because elevated FAS expression is seen in all neoplastic tissues. Moreover, molecular profiling studies by Swinnen *et al* suggest that this biomarker is not only one of the earliest appearing but also one of the most common molecular alterations in prostate cancer (46).

### α-methyl-co-racemase (AMACR)

AMACR is an emerging biomarker which has already achieved clinical acceptance. This protein has been utilized in combination with other cancer markers to visualize infiltration of prostate cancer glands into negative benign prostatic parenchyma facilitating diagnosis (48). AMACR has been utilized in a panel of biomarkers including ERG, GOLPH2 and others to definitively detect early prostate cancer (49).

### GOLPH2

GOLPH2 is a 73-kDa Golgi phosphoprotein of unknown function which has been characterized as a biomarker of prostate cancer (48). Elevated mRNA expression is upregulated in prostate cancer specimens and has been shown to provide greater predictive capacity than PSA (49). Overexpression of GOLPH2 protein has been histologically demonstrated as well, but detection is slightly less sensitive than that of AMACR (48). Laxman *et al* have demonstrated that through the use of urine sedimentation and qPCR early detection of prostate cancer can be determined with greater accuracy than the PSA blood test and >75% positive predictive value. GOLPH2 was incorporated into the multiplex biomarker panel used therein (49).

### Engrailed-2 (EN2)

A subset of genes involved in early embryonic development have been shown to be reawakened during cancer development, notably the *HOX* genes. EN2 is a member of this gene family and has been identified as a transcriptional repressor as well as a translational regulator (50). Investigation into the activation of this gene has yielded a tumor specific biomarker which is secreted by prostate cancer tissue and can be detected in first pass urine (50). Elevated EN2 expression was identified in conditioned media from the prostate cancer cell lines PC3, DU145 and LNCaP, and confirmed in patient biopsies. Development of an ELISA test for detection of EN2 in urine is underway and holds promissing predictive capabilities if confirmed via further investigations.

## 4. Molecular signatures

Just as a fingerprint is unique to each person, cancer cell lines exhibit signature protein pathways, differentiating them from surrounding tissues and other tumors. When these molecular signatures are determined, an individual cancer can be definitively identified, assigned an expected pattern of disease progression, and therapeutically targeted. In theory, this will increase diagnostic accuracy and prolong patient survival. Surprisingly, little research has been invested in examining panels of known prostate cancer biomarkers and their utility. Using existing knowledge, the discovery of novel molecular tumor signatures will enable researchers to diagnose and stage cancer accurately, while opening up a field of selective therapeutics.

### TMPRSS2:ETS gene fusions

The TMPRSS2:ERG chromosomal rearrangement identified by Chinnayan’s pioneering studies in 2005 has become a molecular event of historic proportions in the prostate cancer field. The androgen regulated transmembrane serine protease TMPRSS2 is secreted by prostate epithelial cells in response to ligand exposure and this gene becomes fused with sequences of members of the ETS family of transcriptional activators (ERG, ETV1,4,5). Since TMPRSS2 is expressed in the prostate and regulated by androgens, its fusion to the transcriptional activators ETS gene products could result in driving prostate cancer development, and it appears that this is in fact the case. The prevalence of the fusion products seems to be quite high, although reports vary given that modes of detection vary, different fusion species may exist in a single tumor specimen, and new fusion rearrangements are still being discovered. Regardless, it has been reported that ≥70% of all prostate cancers possess a fusion product (51–57). Despite the functional validation of the prevalence of the fusions, the prognostic value in the clinical setting of prostate cancer patients is still under pursuit. Demichelis and colleagues investigated the impact of the fusion in a watchful waiting cohort of 111 patients and found that those with the fusion had a 2.7-fold increase in prostate cancer-specific mortality compared to those without the fusion, and after 8 years 23% of those without the fusion progressed to metastatic prostate cancer (58). Extensive investigation remains in order to understand the TMPRSS2:ERG gene fusion products role in prostate cancer progression, but it is clear that this molecular event is an early and important marker of prostate cancer. Other groups further investigated the molecular implications of the gene fusion and attempted to identify other genetic prognostic markers in order to develop a panel of genetic signatures that would provide prognostic prediction of biochemical recurrence, based on a cohort of specimens used previously to characterize expression of TMPRSS2:ERG variants (54,59). Using cDNA-mediated annealing selection extension and ligation assay (DASL), 9 upregulated (ERG, HDAC1, ARHGDIB, TRAF4, MSH3, MUC1, YES1, ING1, E2F3) and 6 downregulated genes (CD44, IGF1, MAF, IGFBP6, PTGS1, FZD7) were identified in TMPRSS2:ERG fusion-positive tumors from the aforementioned cohort of samples (59). Using gene ontology analysis, it was determined that mismatch base repair and histone deacetylation functions were over-represented in those genes upregulated with the fusion, and insulin-like growth factor and Jak-Stat signaling pathways in the downregulated genes. These data suggest that there is a unique molecular metabolism functioning in TMPRSS2:ERG fusion-positive tumors. Furthermore, replicating the analysis in a second cohort, Barwick *et al* (59) delineated a set of 9 genes associated with recurrence (CSPG2, CDKN2A, WNT10B, TYMS, E2F3) and non-recurrence (TGFB3, CD44, ALOX12, LAF4) in these patient samples. From these efforts, it was demonstrated that TMPRSS2:ERG fusion status provides a statistically significant predictor of recurrence (P=0.0004), and that the 9 gene panel also yielded a statistically significant predictor of recurrence (P<0.03) (59). The investigation of these clinical and molecular factors provided a molecular signature platform for predicting recurrence in prostate cancer.

### Serine protease inhibitor Kazal type 1 (SPINK1)

SPINK1 is emerging as a biomarker of a molecular subtype of prostate cancer, in the absence of gene rearrangements/fusions such as TMPRSS2:ERG (60). Although long recognized in pancreatic physiology, SPINK1 more recently reemerged as an independent prognostic marker for a variety of cancers, but lacked superiority in predictive value to other commonly used markers (60). Tumors which produce SPINK1 also co-produce activated trypsin. It has been demonstrated that ~10% of prostate cancer cases are SPINK1^+^/fusion^−^ and that this profile can be detected via non-invasive urine assays (61). Furthermore, the SPINK1^+^ outlier expression is a positive predictor of biochemical recurrence after resection, of an aggressive phenotype, and is correlated with Gleason score and poor prognosis (60–62).

New evidence has identified FAS expression as a molecular signature of highly aggressive metastatic prostate cancer. Rossi *et al* characterized unique gene expression profiles that differed significantly between prostate tumors expressing low and high levels of FAS. Increasing FAS protein expression directly correlates with cancer progression (47). In another study, 4 genes, *XLKD1*, *CGA*, *F2R* and *BCL-G*, have been found to reveal cancer recurrence in patients, independent of disease progression (44). An independent analysis of 16 related biomarkers revealed a functional connection with the tumor grade, with each molecular biomarker being assigned to one of five groups based on function. Significantly, it was found that specific composite score for these markers correlated with the Gleason score and cancer staging (63). Moreover, this study firmly established that the composite score was of higher predictive value of cancer grade and relapse than any one of the marker levels alone, confirming the hypothesis that a panel of biomarkers can be more effective for cancer diagnosis than a single marker (63).

Promising advances in biomarker determination have centered around DNA promoter hypermethylation with regard to SLIT2 (64). This epigenetic alteration, well-characterized in a variety of cancer cells, consistently trends toward conserved promoter regions. Gene panels have been explored with fine tuning and those of interest include genes involved in DNA damage and repair, tumor suppressor gene activation, hormonal responses, cell cycle checkpoints and invasion/metastasis. By combining different genes into one panel testing for hyper-methylation, the sensitivity of the panel can be exquisitely enhanced and ultimately refining the discriminatory power of the panel for the exclusive selection of prostate cancer diagnosis. Panels being used currently include combinations of the following genes: *GSTP1* (over-represented in panel compositions), *RASSF1a*, *RARβ*, *APC*, *PTGS2*, *T1G1*, *EDNRB* and *CDH13*, *ASC* (64). This method of hypermethylation detection of genes of interest shows tremendous promise and ability to predict prostate cancer presence and in some cases prognosis and relapse. *GSTP1* methylation has been evaluated independently as a biomarker of prostate cancer n urine sediments, revealing its ability to differentiate between BPH and prostate cancer; furthermore, the frequency of high methylation status correlates strongly with stage III and IV disease (65). The only limitation however for moving such a technique to the clinical setting towards detection of prostate cancer is the small sample sizes analyzed in these studies. Expansion in a considerably larger sample size may establish this technique as a relatively simple and sensitive method to detect prostate cancer.

**Figure 1:**
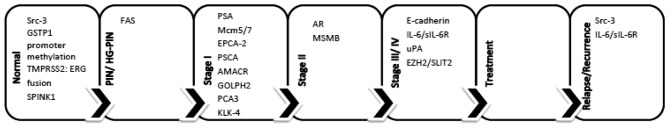
Emerging biomarkers of prostate cancer: continuum of disease progression and stage at which respective biomarkers are anticipated to facilitate clinical utility.

Genome-wide association studies have implicated a handful full of single nucleotide polymorphisms with predisposition to prostate cancer development, specifically in the MSMB gene, which codes for β-microseminoprotein (66). This protein has previously been reported as an early serum biomarker for prostate cancer, but recent refinement has demonstrated that SNPs in the MSMB gene represents a predisposition factor for metastatic prostate cancer (66,67). Advancements of this nature may facilitate our ability to predict the course of disease progression in patients and more aggressively provide treatment based on these prognostic markers. Such studies represent few of the seemingly endless avenues, exploration of which could potentially uncover new diagnostic tools and implement clinical applications. A combination of biomarkers known to indicate prostate cancer proliferation, cell cycle progression, apoptosis loss and signaling of metastasis (anoikis resistance) could be made into a panel of indicative molecules that will present a novel platform of high predictive power of prostate cancer cases. A preliminary panel of emerging biomarkers based on prostate cancer stage is shown in Fig. 1.

## 5. Technology-driven new leads

Recent research has focused on mapping molecular pathways, but the future of prostate cancer research needs to progress in order to use of biomarker panels to detect and characterize tumors. Application of this data can be used to develop novel, tumor-specific treatments, focusing on implementing biomarker panels that will potentially predict the value of interfering with these proteins and their downstream signaling pathways. Information flow within and between cell and tissue compartments through a complex web of biochemical processes provides the ideal forum for biomarker identification. The most prominent features include post-translational modifications of newly synthesized proteins; interactions between kinases and non-enzymatic proteins such as adapters and scaffolds; sequestration within specialized subcellular compartments; intracellular transport; regulated secretion into extracellular space; and assembly, stabilization and disassembly of large, multimeric signaling complexes via ubiquitination and proteasome-mediated degradation. Most of these events are capable of providing critical regulatory control over cell growth, cell survival and apoptosis, anoikis and detachment from the ECM as well as interactions with adjacent cells, neovascularization/angiogenesis and membrane structure and trafficking.

The enormous gaps in our knowledge with respect to clinical pathologies seen in human disease remain. Considering high throughput proteomics data obtained from any physiologic or pathophysiologic situation, one may find that many of the true ‘hits’ have not been previously described at the protein level in any context. In addition, factors in signal transduction mechanisms exhibit a high degree of context-dependence and tissue specificity. Cleverly designed proteomics-based applications in human tissue microarrays from treated and untreated prostate cancer patients, will determine the genes that code for prostate cancer promotion and confer cancer cell survival and resistance to apoptosis and anoikis. Enhanced understanding of the complexity of the molecular mechanisms and expansion of investigative efforts driven by the sophisticated cutting-edge functional genomic and proteomics technology, may result in earlier detection of prostate cancer and more precise staging, and may offer a more accurate prediction and effective management, ultimately resulting in a strong and beneficial impact on patient survival.

## Figures and Tables

**Table I:** Summary of Current Prostate Cancer Biomarkers.

Prostate Cancer Biomarkers in 2011	References
Serum biomarkers	
PSA	
tPSA	(6)
% free PSA	(9)
PSA Density	(10,11)
PSA Velocity	(12)
PSA isoforms	(13)
BPH specific PSA (BPSA)	(14)
Pro-PSA	(14)
Kallikrein-4 (KLK-4)	(15–17)
Steroid Receptor Co-Activator-3 (Src-3)	(16–19)
Minichromosome maintenance 5 protein (Mcm5)	(20,21)
E-cadherin	(22–25)
Early Prostate Cancer antigen	(26,27)
Interleukin 6 (IL-6) and Interleukin 6 Receptor (IL-6R)	(28–30)
Prostate Cancer Gene 3 (PCA3)	(31–33)
Tissue biomarkers	
Urokinase-type Plasminogen Activator (uPA)	(28,34)
Enhancer of Zeste Homolog 2 (EZH2)	(35–40)
Prostate Stem Cell Antigen (PSCA)	(41,42)
Androgen Receptor (AR)	(4,43–45)
Fatty Acid Synthase (FAS)	(46,47)
α-methyl-co-racemase (AMACR)	(48,49)
GOLPH2	(48,49)
Engrailed-2 (EN2)	(50)

## Abbreviations

PSA: prostate specific antigen
tPSA: total prostate specific antigen
AR: androgen receptor
BPH: benign prostate hyperplasia
KLK-4: kallikrein-4
Src-3: steroid receptor coactivator-3
Mcm5/7: minichromosome maintenance protein 5/7
EPCA: early prostate cancer antigen
TNF-α: tumor necrosis factor α
IL-6: interleukin 6
ECM: extracellular matrix
uPA: urokinase-type plasminogen activator
EZH2: enhancer of zeste homolog 2
PSCA: prostate specific stem cell antigen
AR: androgen receptor
FAS: fatty acid synthase
EMT: epithelial-mesenchymal transition
AMACR: α-methyl-co-racemase
GOLPH2: golgi protein H2
EN2: engrailed-2
